# Central Object Segmentation by Deep Learning to Continuously Monitor Fruit Growth through RGB Images

**DOI:** 10.3390/s21216999

**Published:** 2021-10-21

**Authors:** Motohisa Fukuda, Takashi Okuno, Shinya Yuki

**Affiliations:** 1 Faculty of Science, Yamagata University, 1-4-12 Kojirakawa, Yamagata 990-8560, Japan; okuno@sci.kj.yamagata-u.ac.jp; 2 Elix Inc., Daini Togo Park Building 3F, 8-34 Yonbancho, Chiyoda-ku, Tokyo 102-0081, Japan; shinya.yuki@elix-inc.com

**Keywords:** deep learning, U-Net, image segmentation, central object, fruit, pear, growth monitor, RGB images

## Abstract

Monitoring fruit growth is useful when estimating final yields in advance and predicting optimum harvest times. However, observing fruit all day at the farm via RGB images is not an easy task because the light conditions are constantly changing. In this paper, we present CROP (Central Roundish Object Painter). The method involves image segmentation by deep learning, and the architecture of the neural network is a deeper version of U-Net. CROP identifies different types of central roundish fruit in an RGB image in varied light conditions, and creates a corresponding mask. Counting the mask pixels gives the relative two-dimensional size of the fruit, and in this way, time-series images may provide a non-contact means of automatically monitoring fruit growth. Although our measurement unit is different from the traditional one (length), we believe that shape identification potentially provides more information. Interestingly, CROP can have a more general use, working even for some other roundish objects. For this reason, we hope that CROP and our methodology yield big data to promote scientific advancements in horticultural science and other fields.

## 1. Introduction

The following Figure 1 gives a quick overview of CROP the neural network to be introduced in this paper. Counting the pixels belonging to the masks, which are represented by red color, gives the relative sizes of the fruit. Applying this method to time-series images captured by fixed cameras, one can keep track of the fruit growth.

Please note that USDA ARS stands for United States Department of Agriculture, Agricultural Research Service, and the photos were obtained from their image gallery. Use of photos of USDA ARS is not meant to infer or imply USDA ARS endorsement of any product, company, or position. The original photos were cropped and processed.

### 1.1. Monitoring Fruit Growth

Techniques of computer vision have many applications in fruit production, for example, making yield estimates by detecting fruit in the farms [1]. Non-contact size measurements and shape description are also useful in making yield estimates and distribution plans of fruit [2,3,4]. Indeed, in [5] using Bertalanffy growth model, predictability of fruit size at the time of harvest based on the measurements at the earlier development stages was discussed. Furthermore, when change of colors cannot be a measure of ripeness, size and volume growth can be good candidates for indexes regarding such predictions; see Section 1.4 for the pear production in Japan. In fact, devices to continuously measure fruit size have been developed, for example stainless frames with potentiometers to be put on fruit [6], and flexible tapes around fruit to be read by infrared reflex sensors [7].

Recently deep learning techniques are applied to monitor growth of mushrooms [8] and apples [9]. These two results share the same spirit with ours in the sense that deep neural networks are used to process images captured by fixed cameras to acquire the time-series size data. The former was conducted in the greenhouse with the light source at the top and the images were captured from fixed cameras above, which probably offered stable light conditions. These images were then processed by the object-detection deep neural network YOLOv3 [10] to locate mushroom caps, and next by a so-called SP algorithm to calculate the diameters. In the latter, the neural network was built upon ResNet-50 [11] a popular image-classification deep neural network. Then, the images of apples in the orchard captured on cloudy days or at dusk were processed by a Laplace operator and then by the deep neural network, to detect the edges to predict the diameters.

### 1.2. Image Segmentation and Our Model

Initially, computer vision methods for fruit yield estimate are based on pixel-wise color analysis, for example, in [12] fruit pixels were counted to obtain the occupancy ratios in the images using the thresholds on red, green and blue. Later, techniques of so-called *image segmentation* started being applied; image segmentation means classifying image pixels into segments. With such techniques, one can isolate fruit pixels in images and count the number of blobs for the yield estimates. Such examples include [13,14], and [15] (with the controlled illumination at night). Additionally, techniques of *machine learning* were applied, for example *X-means clustering* in [16] and *conditional random field* for multi-spectral data in [17].

Moreover, image segmentation techniques have also been applied to non-contact size and volume measurement of fruit, or more broadly shape description. Such examples include: via Hough transform [18], using two cameras [19], and on smart phones [20]. In [21], a machine learning method called *support vector machine* was applied.

Many of deep learning methods applied in horticultural science fall in the category of object detection, with which one can obtain bounding boxes (usually rectangles) to locate objects in images. Faster R-CNN [22] is one of famous DNNs for object detection, and yielded successful applications for example in detecting apples [23], mangoes with spatial registration [24], and sweet papers using multi-modal data (RGB colors and Near-infrared) [25]. However, such rectangular bounding boxes are not descriptive enough to obtain the sizes or shapes of the target fruits. Indeed, in [8] applying YOLOv3 [10] was not enough, as described above. Now, Mask-RCNN [26] is an implementation of instance segmentation, which locates individual objects and applies image segmentation accordingly. It was applied to identify individual bunches of grapes for more accurate size (volume) estimates in [27], and to identify individual blue berries for maturity estimations in [28]. Moreover, in [29] DNNs were trained by synthesized data to directly count tomatoes.

For more accurate size measurement and shape description, one needs to put more weight on image segmentation. Recently, deep learning methods started replacing some of the pixel-wise color analysis methods for image segmentation in locating and counting fruit [30] (see [31] as well). As discussed above, in [9] then deep learning was applied for edge detection, which is closely related to image segmentation, in order to predict the diameters of apples. Although there are preferred light conditions and necessary prepossessing in [9], our neural network, which we call CROP (Central Roundish Object Painter), conducts image segmentation and makes masks for various roundish fruit located in the center of un-preprocessed images captured in varied surroundings; even with near-infrared light flash in the dark. Although counting the pixels of these masks gives the relative 2-dimensional size of the fruit, which is different from the conventional measures of fruit size, one could keep track of the pear growth as in Section 3.4 and Section 3.5. Moreover, we believe that this new measure is more stable against measurement disturbances. Furthermore, the methodology would be easily transferred to other types of roundish fruit, which was qualitatively examined in Section 3.3.

### 1.3. About Deep Learning

Deep learning (DL) is one of machine learning techniques. There are more and more applications of DL not only in fruit production but also in agriculture [32]. Now, we briefly look into DL. Human-made algorithms require humans to set features to be extracted from raw data. In reality, fruit images in farms vary extensively. Indeed, the fruit can be of any species and at any development stage. Even if these variables are fixed, the surroundings can change depending on time of day, weather conditions, seasons, and other illumination conditions such as shade and light reflection. Therefore, potentially huge number of features are necessary in acquiring desired information from such images. In this case, then it may be simply too much for humans to find and list all important features to write algorithms by hand. In contrast, data-driven methods, such as DL with DNNs (Deep Neural Networks), could extract important features automatically, so that they process data in a ‘black box’, although humans can guess how they process data using for example Grad-CAM [33]. Roughly writing, as data flow in a DNN made of many layers, each layer makes the data a little more abstract, so that the DNN yields abstract understanding deep within itself. This way, humans may not have to pick important features by themselves, which distinguishes DL from conventional methods. Interested readers can consult [34] for more detailed explanations on DL.

### 1.4. Pear Production in Japan

*La France* is one of the most popular cultivars of European pear (Pyrus communis) in Japan, its yield is about 70% of European pears in Japan. La France pomes are usually harvested at the mature-green stage and then chilled for stimulation of ethylene biosynthesis prior to being ripened at the room temperature. If the harvest is delayed or too early, the fruit does not ripe properly, and the texture, taste and flavor will be poor. In commercial pear cultivation, harvest time of La France greatly influences both the amount and quality of the harvest. Therefore, it is important to measure the maturity of the fruit precisely to optimize the time to harvest, but criteria to estimate the fruit maturity are limited such as fruit firmness and blooming date. The fruit growth in terms of fruit size is described by an asymmetric sigmoid curve. The growth rate of the fruit on the tree is significantly affected by environmental factors and the physiologically active state. Precise time-lapse measurement of fruit growth should be useful for estimation of the fruit maturation status. To measure the fruit size change as it grows, the size of the same fruit must be measured repeatedly (daily) with a caliper. However instead, we propose to record the area change of the fruit in the time-series images captured by fixed cameras. We hope that our methodology provides horticultural scientists with big data on fruit development processes with least labor.

## 2. Methods and Materials

### 2.1. Neural Networks for Image Segmentation

In a sense, *image segmentation* can be seen as classification of image pixels. For example, in this study, we want to cut out the central fruit in an image, but it amounts to deciding which class label each pixel belongs to, the central fruit or the region outside it, i.e., the background. In general, the number of class labels can be more than two and such image processing is called *semantic segmentation*. In the rest of this section, we explain about our neural networks and compare them with others.

Strictly speaking, CROP is the name for our trained DNNs with special properties, but we call them so even before the training for convenience. As in Figure 2a, CROP takes in 512×512-pixel RGB images and outputs 512×512-pixel gray-scale images. These inputs and outputs are normalized to take decimal numbers between 0 and 1, unlike usual images taking integer values between 0 and 255. The outputs will turn into masks with some threshold, for example 0.5 in our case. The downward red arrows represent convolution layers with kernel 2×2 and stride 2, which double the number of channels. Here, *channel* refers to another dimension than height and width. For example, the number of channels of RGB images is 3 and that of gray-scale images is 1. Similarly, the upward green arrows represent transposed convolution layers with kernel 2×2 and stride 2, which however make the number of channels half. The red rectangles are concatenations of two convolution layers with kernel 3×3 and stride 1, which keep the number of channels unchanged. The green rectangles are again concatenations of convolution layers, where the second convolutions are the same as the ones from the red rectangles, but the first ones are a bit different. Their inputs are direct sums (in the channel space) of the outputs of the layers below and those from the left, the latter of which are indicated by horizontal arrows. With these inputs then those concatenated convolution layers make the number of channels half. Pink and light green boxes represent again concatenations of convolution layers with kernel 3×3 and stride 1. Going through these layers the number of channels changes as 3→16→16 and 32→16→16→1, respectively.

The architecture of CROP is based on U-Net [35]. U-Net was developed for medical image segmentation, and many related research projects were conducted, including V-Net for 3-dimensional medical images [36], from which we adopted the loss function. U-Net (and V-net as well) belongs to the family of *Convolutional Neural Networks (CNNs)*, which treat 2-dimensional images as they are. Some argue that CNNs can be traced back to [37]. Historically, CNNs proved to be useful for example in handwriting recognition [38], and then AlexNet [39] improved the error rate dramatically at ImageNet Large Scale Visual Recognition Challenge in 2012. Naturally, this architecture started being adapted to semantic segmentation for example in [40], and with *encoder-decoder* structure in [41,42]. The encoder-decoder structure consists of two parts; in addition to size-invariant convolutions, encoding with convolutions or max pools and decoding with transposed convolutions or up samplings. The encoder extracts image features and is sometimes called *backbone*. Indeed, CNNs trained for image classification, which we suppose have already learned how to extract features, are often placed as backbones, for example ResNet-50 [11]. In Figure 2a, the left hand side corresponds to the encoder and the right the decoder. Importantly, there are *skip connections* represented by horizontal arrows. They are supposed to transmit location information from the encoder to the decoder, and characterize U-Net.

CROP has a similar structure as U-Net, but the decoder and encoder are deeper than those of U-Net. In this study, CROP-Shallow in Figure 2b can be thought to be analogous of U-Net. Indeed, U-Net applies max pools 4 times, and at the bottom the number of channels will become 1024 while the size of feature map will be nearly $2^{-4}$ as small as the input image. In contrast, CROP applies 7 times convolution operations with kernel 2×2 and stride 2, and at the bottom the size of feature map will be $2^{-7}$ as small as the input image. We believe that this difference in depth enables CROP to have larger receptive fields; see Section 3.2 for quantitative experiments on this matter.

Before concluding this section, we write about two more well-known DNNs for image (instance) segmentation. First, Mask R-CNN [26] is an implementation of *instance segmentation*. Instance segmentation distinguishes individual instances of the same class label, while semantic segmentation does not. Although it can have wide-ranging applications in horticultural science (see Section 1.3 for some), since we do not have to analyze all fruit in the image, we can rather focus on image segmentation. Next, DeepLabv3+ [43] is an implementation of semantic segmentation and has *atrous convolutions* to capture contextual information. However, because we do not need contextual information for our purpose, we chose to use U-Net for preciser image segmentation.

### 2.2. Loss Functions and Evaluation Criteria

Choice of loss functions defines how to update the parameters of DNNs, i.e., how they learn. In this project, we used *soft dice loss* [36]:

(1) $$1-2\left(\sum_{k}x_{k}t_{k}\right)/\left(\sum_{k}x_{k}^{2}+\sum_{k}t_{k}^{2}\right)$$

which is a variant of *dice loss*, where *k* runs over all the pixels; 512×512 in our case. Additionally ${\left\{x_{k}\right\}}_{k}$ are outputs of CROP (gray-scale images), taking values between 0 and 1 after going through the sigmoid function: $1/(1+e^{-x})$, while ${\left\{t_{k}\right\}}_{k}$ are ground truth (masks), taking values of only 0 or 1, where 0 and 1 correspond to the background and the central roundish object, respectively; we swapped the roles and took the average. Please note that the loss vanishes if $x_{k}=t_{k}$ for all *k*’s. Of course, pixel-wise *cross-entropy*: $\sum_{k}-t_{k}\log x_{k}-\left(1-t_{k}\right)\log\left(1-x_{k}\right)$ may seem to be a good choice if we consider image segmentation as classification of each pixel; cross-entropy is commonly used for image-classification tasks. However, with cross-entropy each pixel would carry the same share in the loss. Consequently, each mis-classified pixel would make the same amount of contribution to back-propagation (training process) regardless of the size of objects, and then it could let DNNs learn to rather ignore small objects. In contrast, with soft dice loss such imbalance would be compensated by regularization, which appears as the denominator in (1). For similar reasons, we did not use $l_{p}$ loss (p≥1): $\sum_{k}{\left(x_{k}-t_{k}\right)}^{p}$.

Finally, *IoU (Intersection over Union)*, or *Jaccard index*:

$$\mathrm{IoU}\left(A,B\right)=\frac{\left|A\cap B\right|}{\left|A\cup B\right|}\;.$$

measures how two sets are close to each other. It takes values between 0 and 1; A∩B=∅ and A=B give 0 and 1, respectively. This is not a loss function because it is not differentiable, but was used in this paper to compare masks made by CROP and the ground truth masks to evaluate CROP.

### 2.3. Datasets

In this project, we used three groups of images downloaded from the Internet and captured at the farms (Kaminoyama, Yamagata, Japan).

Data_Fruit 172 images of various fruit downloaded from Pixabay:

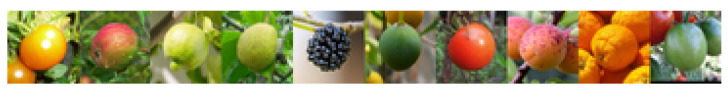

Data_Pears1 26 images of pears at the farm in 2018 with Brinno BCC100 (time-lapse mode):

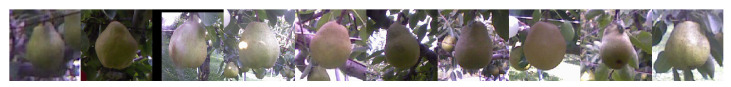

Data_Pears2 86 images of pears at the farm in 2019 with various cameras:

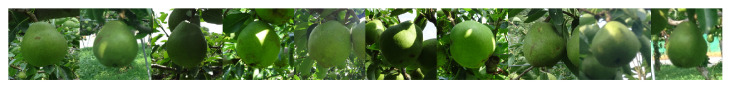

These images were all annotated by labelme [44] to train and evaluate CROP. Additionally, Brinno BCC100 is a time-lapse camera previously used by one of the authors to keep track of the development process of pears.

For training and evaluation, data augmentation was conducted, unless otherwise stated. It includes geometrical changes: size alternations, horizontal and vertical flips, rotations, and various photo-metric changes.

### 2.4. Hardware and Software

For this project, the following two GPUs were used: TITAN Xp and GeForce RTX 2080 Ti. The machine learning library was PyTorch [45]. The graphs in this paper were drawn by Matplotlib [46] and Seaborn [47].

## 3. Results

### 3.1. Quantitative Analysis

#### 3.1.1. Training CROP

For quantitative analysis we divided Data_Fruit randomly into the training dataset (80%: 137) and the validation dataset (20%: 35). Initial parameters of CROP were set randomly, where we did not use a pre-trained model for the backbone. The parameters were then updated by Adam with learning rate 0.001. The result can be seen in Figure 3, where the best IoU for the validation data were 0.984, achieved at the epoch 9700, where the threshold for masks was 0.5. In this experiment, The individual evaluations were made every 100 epochs and were the average over the 5 independent evaluations with application of random data augmentations, i.e., the training dataset and the validation dataset were practically of 137×5 and 35×5, respectively. To obtain an idea about the IoU, suppose that we have two 100-pixel masks of the ground truth and a prediction, and that 99 pixels are correctly predicted. Then, the IoU would be:

$$\mathrm{IoU}(\mathrm{ground}\;\mathrm{truth},\;\mathrm{prediction})=\frac{99}{101}=0.98019\ldots$$

This optimal CROP corresponds to the network dictionary named net_dic_0601_09700, and was applied to test data (Data_Pears1) in Section 3.4 to give predictions before the fine-tuning. Please note that the network dictionaries store the learned parameters of the neural networks. On the other hand, for qualitative analysis in Section 3.3, CROP was trained by all the images of Data_Fruit until epoch 5000. The corresponding network dictionary was named net_dic_0314_05000. These two dictionaries are available on GitHub.

#### 3.1.2. Fine-Tuning CROP

For fine-tuning, we divided Data_Pears2 randomly into the training dataset (80%: 68) and the validation dataset (20%: 18), and then we trained further the optimal CROP from Section 3.1.1 with the dictionary named net_dic_0601_09700. The optimization method was Adam with learning rate 0.0001. One can see the process in Figure 4; the best IoU for the validation data was 0.983 achieved at epoch 5,100. This CROP was applied to test data (Data_Pears1) in Section 3.4 to provide predictions after the fine-tuning. This network dictionary was named net_dic_ft_1015_05100. Furthermore, we fine-tuned the network dictionary named net_dic_0314_05000 for 5000 epochs using the whole Data_Pears2 to obtain the network dictionary named net_dic_ft_0328_1_5000, which was used in Section 3.5. These two dictionaries are also available on GitHub. One can see the improvement of the IoU in Table 1, and the refinement in some examples of test data (Data_Pears1) in Section 3.4. Note that data augmentation was not applied in the evaluation.

### 3.2. Depth of Neural Networks

In this section, we report on experiments on CROP-Shallow (Figure 2b). We trained CROP-Shallow and CROP in the same conditions: loss function, optimization method, learning rate and batch size, i.e., they were the same as in Section 3.1.1 except that the batch size was 6, which is the maximum for CROP-Shallow in terms of the GPU memory capacity. Random were partitions of training and validation datasets, initialization of these neural networks, batches and application of data augmentation. In the same way as Section 3.1.1, random data augmentation was applied to have 137×5 training dataset and the 35×5 validation dataset for the individual evaluations. These random methods were adopted to avoid particular biases, and moreover three independent experiments were performed for each neural network. One can see from Table 2 and Figure 5 that CROP fits our dataset more and the IoUs of CROP-Shallow seem to have hit the ceiling towards the epoch 2500. We believe the difference came from the fact that segmenting larger objects in images would require larger receptive fields, which can be obtained from deeper CNNs (remember convolution layers act locally), while U-Net was originally invented to segment rather small cells in biomedical images.

### 3.3. Qualitative Analysis

Now, we apply CROP to sample images. To make each prediction stable, we fed CROP the images made by the eight transformations composed by flips and rotations, which keep a right square unchanged in the two-dimensional space. Then, each mask was made from the average of those eight outputs with threshold 0.5. In the figures, the masks were pasted onto the input images; red for the central object and yellow for the background. The implementation of this image processing is also available on GitHub. Please note that the sample images in this section do not belong to our datasets, and they can be thought of as test data, i.e., they are new to CROP.

First, examples in Figure 6a,b,d indicate how CROP identified individual central grapes. In contrast, CROP became confused in Figure 6c because the central grape was behind others. Next, one can see in Figure 7 that CROP handled images of various fruit, dealing with peduncle and calyx. However, unsuccessful examples are presented in Figure 8. Finally, it is interesting to point out that although CROP has been trained solely by 172 fruit images, without transfer learning, it gained some sort of generality as in Figure 9.

### 3.4. Fine-Tuning to Pears

In this section, we make qualitative analysis of fine-tuning explained in Section 3.1.2. In Figure 10, each triple consists of the original image, and processed images before and after the fine-tuning, placed from left to right.

### 3.5. Applying CROP to Time-Series Images

In this section, we demonstrate our methodology by processing 510 images on the target pear, which were captured in Kaminoyama, Yamagata, Japan during 12 August 2020 13:49–15 October 2020 8:00; eight a day at 8:00, 9:49, 11:49, 13:49, 15:49, 17:49, 19:49, 21:49, except for the first five and the last one. The images were given IDs from 2 to 511 chronologically. The camera was SINEI SC-MB68 a trail camera. Please note that these images are new to CROP; see Section 2.3 for our datasets. At the end of this section, we treat only the daytime images and remove outliers based on the coefficient of variation (standard deviation divided by the mean) to obtain a growth curve of the pear.

**Figure 11:**
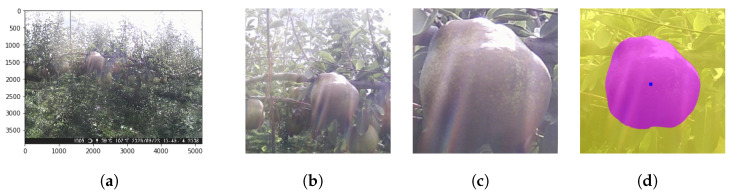
(**a**) The original image of 5200×3900 pixels. (**b**) Choosing the target fruit roughly manually. (**c**) CROP will then recognize the central fruit. (**d**) It will measure the area as the number of pixels of the mask and give the 2D-wise center of mass. The pixel number is re-scaled in the scale of (**a**) and hence is in general decimal. See Figure 12 for how to obtain the mask. The image ID is 338.

**Figure 12:**
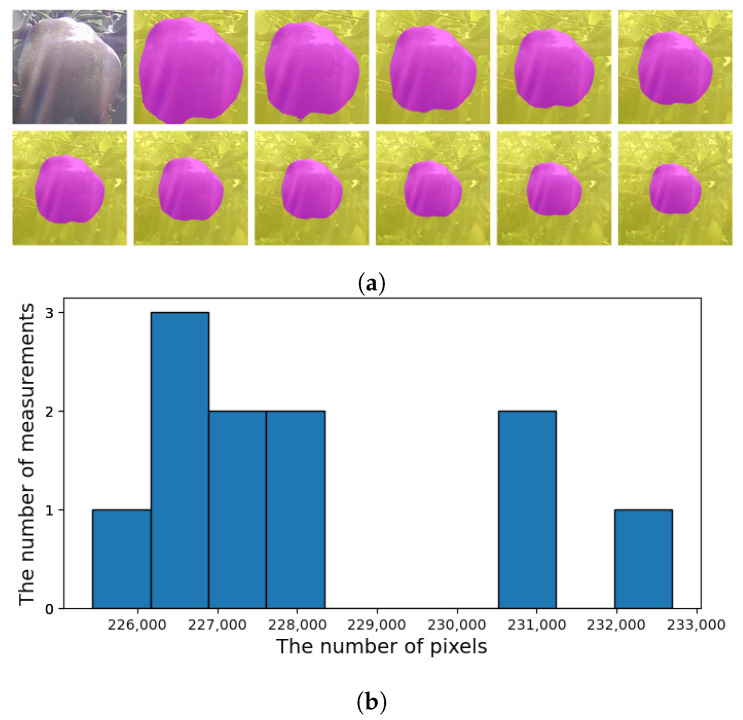
(**a**) CROP makes 11 measurements in different scale factors; from ×1.0 to ×0.5. (**b**) The median of re-scaled values will be picked to exclude outliers. The corresponding mask will be picked as in Figure 11d. The image ID is 338.

**Figure 1:**
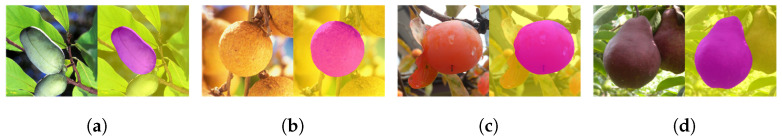
CROP identifies and paints central fruit of various kinds. These images were not used for the training or validation, and are considered to be test data. (**a**) pawpaw. (**b**) longan. (**c**) persimmon. (**d**) pear. The original photos of (**a**,**b**) are credited to USDA ARS (Scott Bauer), and those of (**c**,**d**) Hideki Murayama.

The key ideas in processing time-series images by CROP are:

1. It can detect the central roundish fruit as in Figure 11b,c. By applying this functionality repeatedly, it can keep track of the target fruit.
2. It can work in different scales. One can take the median of 11 measurements of different scales to exclude outliers as in Figure 12.
3. It can keep track of the 2D-wise center of mass of the mask, as in Figure 11d.

The graph in Figure 13 shows the growth curve of the target fruit where the alleged outliers remain (some are out of the range). The growth curve here is estimated by the areas of the masks created by CROP; taking the square root may give the equivalent measure for the diameter or length, though.

Now, we shall focus on the five days: 8–12 October 2020 (the image IDs are from 455 to 494), where the measurements were rather stable. Figure 14 shows the boxplot of all the measurements. Each box represents the 11 measurements as in Figure 12. One can see that the measurements tend to have higher variance during the evening time (17:49, 19:49, 21:49). In addition, the eight images captured on 12 October 2020 and their predicted masks are in Figure 15.

Also, Figure 16a shows how the target fruit moved around during the whole season, where some possible outliers stay inside and outside the range. On the other hand, Figure 16b describes the positions of the target fruit during the above five days.

To obtain a nicer growth curve, we treat only 315 daytime images; 5 images per day for 63 days during 13 August–14 October 2020. We used coefficient of variation (CV), which is the standard deviation divided by the mean, to identify outliers; the statistics are made of the 11 measurements (Figure 12). More concretely, we replaced the medians of the 15 images with largest CVs, which amount to less than the 5% of 315, by the medians of the previous images. The histograms of the top 15 CVs and others are found in Figure 17.

In Figure 18a,b, one can see the processed images corresponding to the two highest CVs. Additionally, we noticed that there was a confident mistake for the ID 393 (Figure 18c), which stood out in the provisional plotting and was modified manually in the same way. After applying these changes, we obtained the growth curve shown in Figure 19. All the boxplots after the modification are placed in Appendix A. The 16th largest CV corresponds to the ID 338, which was used in Figure 11 and Figure 12. The outliers appearing at ID 338 of Figure A6 was properly dealt with as in Figure 12; these two sets of measurements were conducted independently, though.

Finally, we shall emphasize the fact that it took only 791.1025 s, which is less than 14 min, to process the 510 images with NVIDIA TITAN Xp. All the process was automatic after specifying the target fruit similarly as in Figure 11b for the ID 2. During this process, for individual images, CROP made the 11 measurements and chose the median and calculated the center of mass, so that it saved all the numerical data as csv files and all the masks as png files.

## 4. Discussions

**Figure 2:**
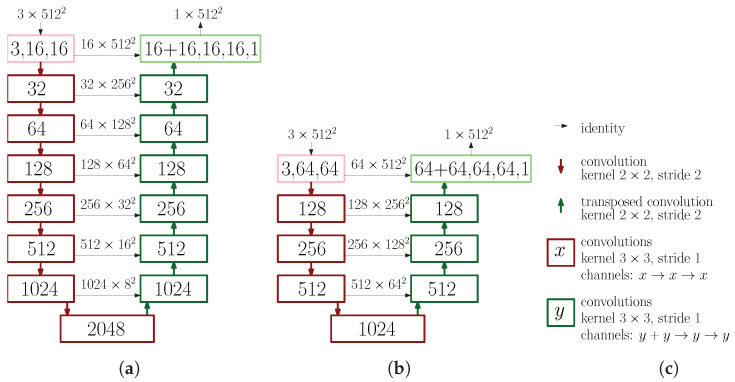
The architectures the deep neural networks. (**a**) CROP. (**b**) CROP-Shallow. (**c**) Notations. ReLU and *batch normalization* are applied adequately, which are not explicit in the figure.

**Figure 3:**
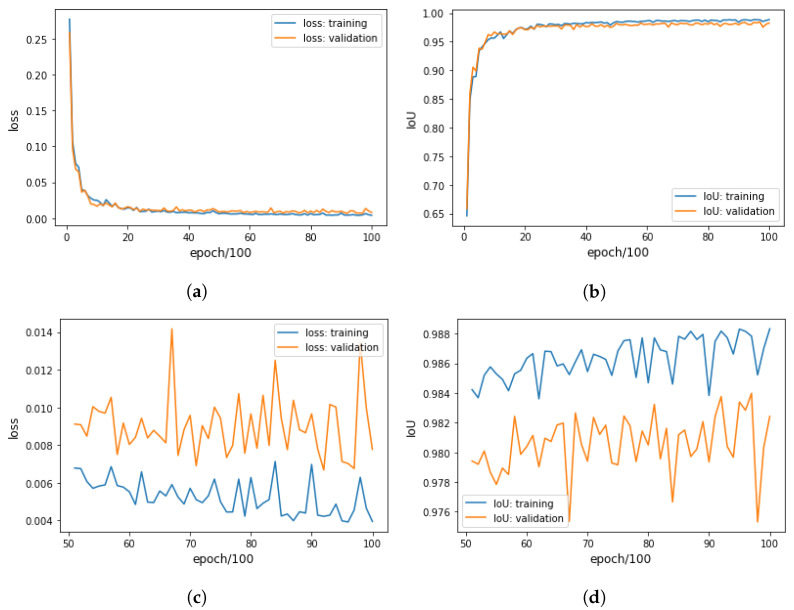
Training CROP with the training dataset (80%: 137) and the validation dataset (20%: 35) of Data_Fruit, so that the best IoU for the validation data was 0.984 at epoch 9700. (**a**) Losses for epoch 100–10,000. (**b**) IoUs for epoch 100–10,000. (**c**) Losses for the last half; epoch 5100–10,000. (**d**) IoUs for the last half; epoch 5100–10,000.

**Figure 4:**
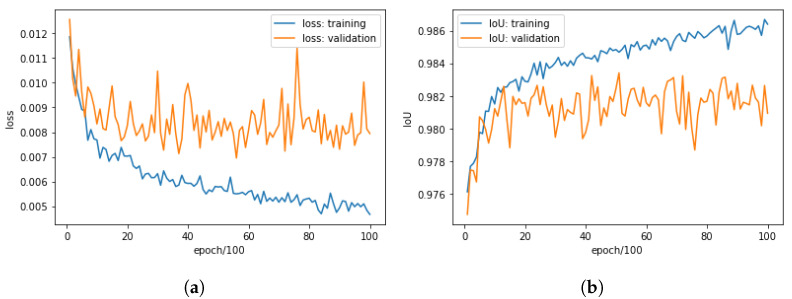
Fine-tuning CROP with the training dataset (80%: 68) and the validation dataset (20%: 18) of Data_Pears2, so that the best IoU for the validation data was 0.983 achieved at epoch 5100. (**a**) Losses for epoch 100–10,000. (**b**) IoUs for epoch 100–10,000.

**Figure 5:**
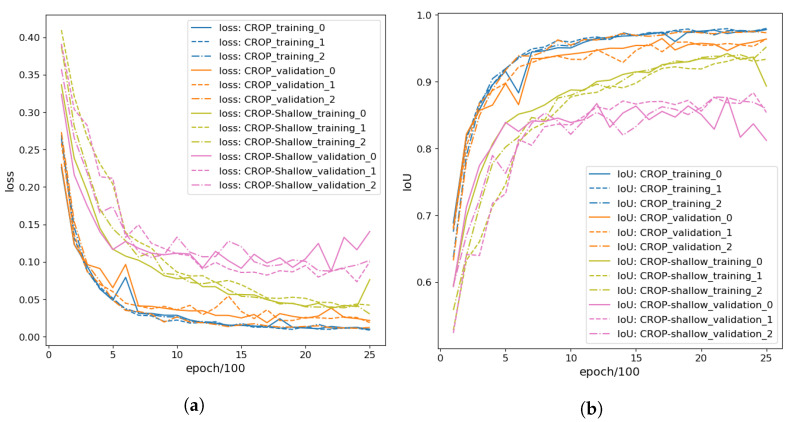
Comparing the training processes of CROP and CROP-Shallow through three experiments for each, numbered from 0 to 2. They were conducted for 2500 epochs with the training dataset (80%: 137) and the validation dataset (20%: 35) of Data_Fruit. (**a**) Losses for epoch 100–2500. (**b**) IoUs for epoch 100–2500.

**Figure 6:**
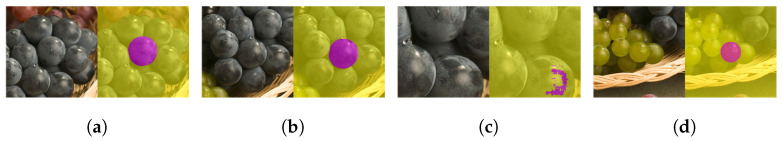
Identifying the central grapes in the bunch of grapes. (**a**) The central grape is identified. (**b**) The central grape in another frame is identified. (**c**) CROP is confused because there is no central fruit in front. (**d**) The central object can be small, and may not have to be salient. The original photo is credited to USDA ARS (Peggy Greb).

**Figure 7:**
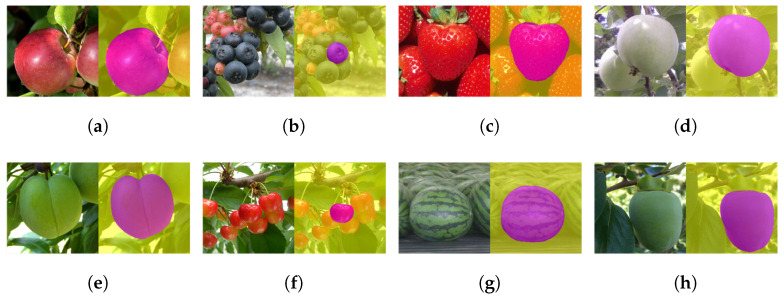
Examples (**a**–**h**), where peduncle and calyx are properly ignored. Some of the original photos are credited to USDA ARS; (**a**) to Peggy Greb, (**b**) to Mark Ehlenfeldt, (**c**) to Brian Prechtel. Moreover, the original photos of (**e**–**h**) are credited to Hideki Murayama.

**Figure 8:**
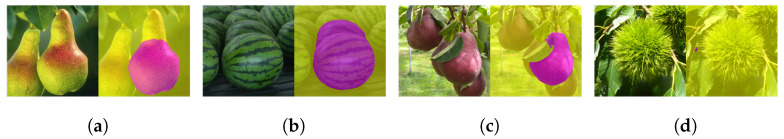
Unsuccessful examples. (**a**) The central fruit is not round enough. (**b**) Two boundaries were mixed up. (**c**) The central object is disrupted by the leaf. (**d**) The boundary is spiky. The original photo of (**a**) is credited to USDA ARS (Keith Weller). The original photos of (**b**–**d**) are credited to Hideki Murayama.

**Figure 9:**
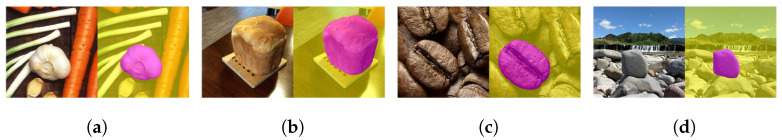
Gained generality and works for various objects. (**a**) A garlic. (**b**) A loaf of bread. (**c**) A roasted coffee bean. (**d**) A stone. The original photo of (**a**) is credited to USDA ARS (Scott Bauer).

**Figure 10:**
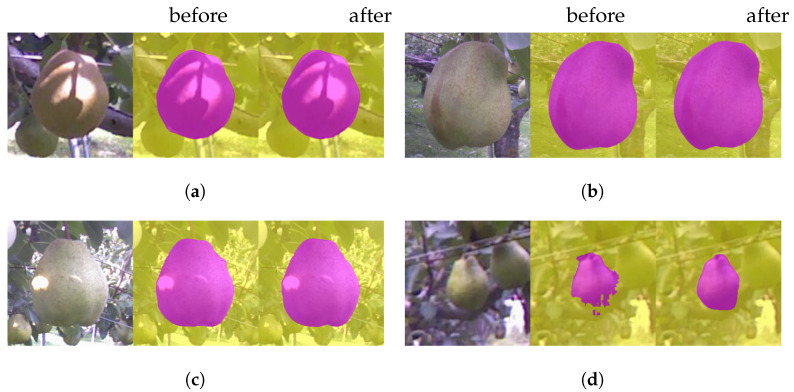
Before and after the fine-tuning. (**a**) This image was properly processed even before the fine-tuning. (**b**) The bottom contour line became more precise. (**c**) The right boundary became more accurate. (**d**) A good amount of improvement.

**Figure 13:**
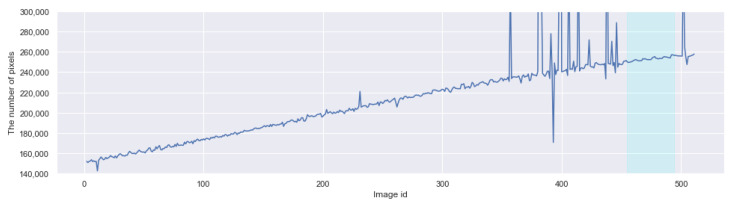
The number of pixels of the masks predicted by CROP for the period 12 August–15 October 2020. Outliers are plotted inside and outside the range. The five days highlighted by cyan color is treated separately in Figure 14.

**Figure 14:**
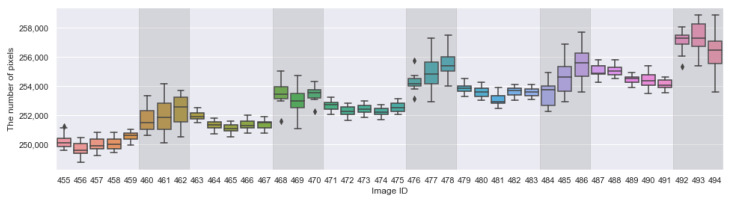
The box-plot of the 11 measurements (see Figure 12a) during the period 8–12 October 2020. The variance is higher in the evening (17:49, 19:49, 21:49), indicated by the darker background. The higher the variance, the longer the box and the whisker, and the more individual the points. The horizontal line in each box represents the median. The images captured on the last day (from 487 to 494) are found in Figure 15.

**Figure 15:**
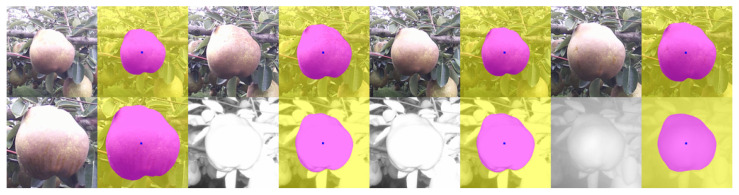
The eight images on 12 October 2020, the last day in Figure 14, whose image IDs are 487 to 494. These masks gave the medians appearing as part of the graph in Figure 13 and in Figure 14. Additionally, the blue dots describe the relative positions, which constitute part of the dots in Figure 16.

**Figure 16:**
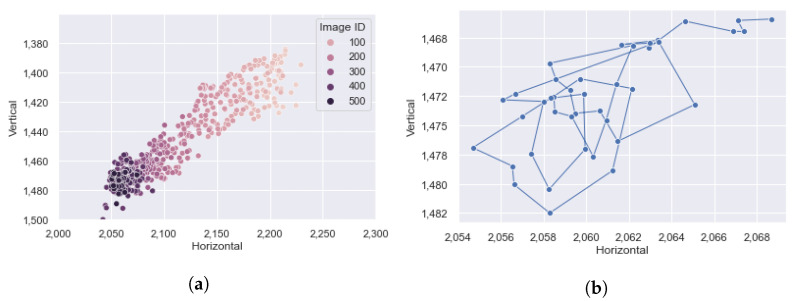
The positions of the target fruit, predicted by CROP. The coordinates are pixel numbers in the original image (Figure 11a), counting from the top-left corner. (**a**) All the positions were plotted including possible outliers inside and outside (to the below of) the range. The darker the color is, the later the image was captured. (**b**) Visualization of the movement during the five days: 8–12 October 2020.

**Figure 17:**
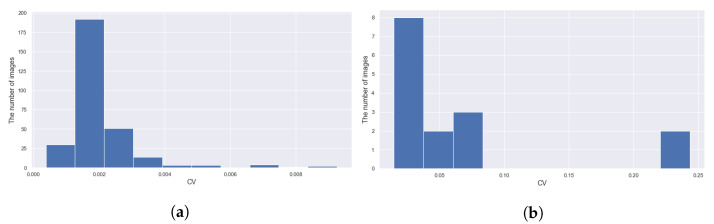
The histograms of CVs of the 11 measurements for individual images (Figure 12). (**a**) The 300 smaller CVs, which we consider as normal. (**b**) The 15 largest CVs, which we treat as the evidence for outliers.

**Figure 18:**
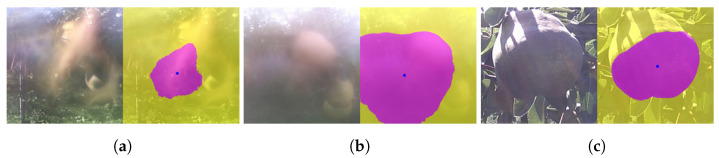
Examples of images with the highest CVs and a confident mistake. (**a**) This blurred image gave the highest CV (id: 381). (**b**) The misty weather condition resulted in the second highest CV (id: 399). (**c**) The unsuccessful case modified manually (id: 393).

**Figure 19:**
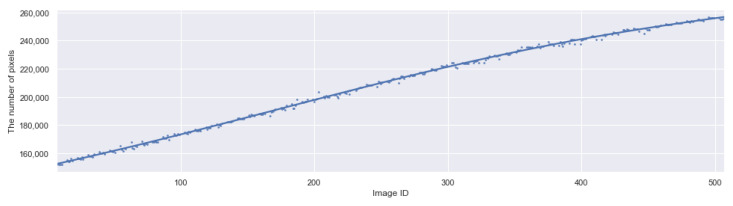
The plot of the modified data and the growth curve made by curve-fitting to 5th degree polynomials, with Seaborn [47].

**Figure A6:**
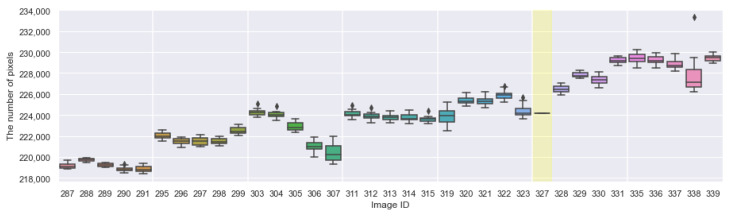
17–23 September.

In this project, CROP had gained general ability to segment central roundish objects, although we trained it by fruit images. With more training data, it would increase accuracy and generality. Note however that the image processing of CROP is different from *salient object detection* [48], as CROP identifies a small central object such as the one in Figure 6d. This is why we call our method *central image segmentation*. We hope that our non-contact method of size measurement will provide scientific big data for the advancement of science, especially in the field of fruit production. In fact, as of September 2021, pear cultivators in Kaminoyama Yamagata can know, via the smartphone application, the daily sizes of the sampled pears, which are predicted by CROP. To conclude this paper, we briefly write how one can adapt CROP to multi/hyper-spectral camera images. In principle, if you have *n*-band images, the number 3 in the pink box in Figure 2a should be replaced by *n*. For example, thermal cameras result in n=1 and RGBN cameras n=4. How large *n* can be depends on how much memory the GPU has. Our GPU TITAN Xp has 12GB of memory, and in this case we believe *n* could be 10 or so while the training may become unstable because the batch size would be smaller. This problem can be overcome to some extent conducting parallel computation which can deal with batch normalization problem. Apart from the GPU memory issue, we believe when *n* is large one should think whether or not at least the channel number 16 in the pink box in Figure 2a is appropriate, because *n*-mode images, i.e., *n*-dimensional information, would be mapped into 16-dimensional space very at the beginning.

## Figures and Tables

**Table 1:** IoUs for Data_Pears1 before and after the fine-tuning with Data_Pears2.

	Before	After
IoU	0.882	0.917

**Table 2:** The best IoUs for the validation datasets with CROP and CROP-Shallow, with epoch not more than 2500, using the training dataset (80%: 137) and the validation dataset (20%: 35) of Data_Fruit. Three experiments, numbered from 0 to 2, were performed for each.

	CROP			CROP-Shallow		
experiment	0	1	2	0	1	2
best IoU	0.965	0.964	0.975	0.876	0.884	0.877

**Figure A1:**
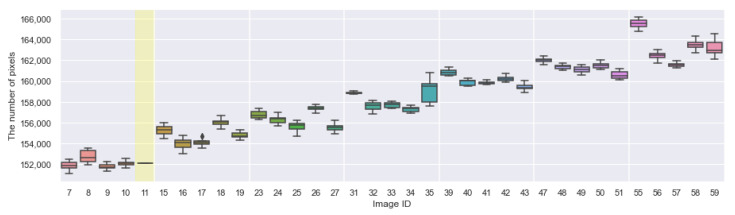
13–19 August.

**Figure A2:**
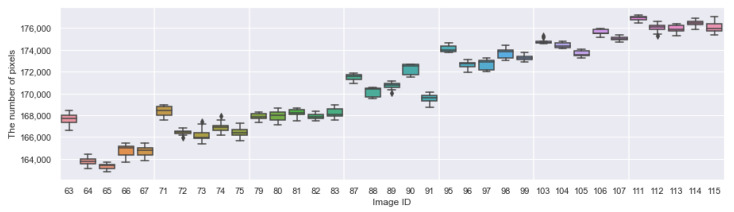
20–26 August.

**Figure A3:**
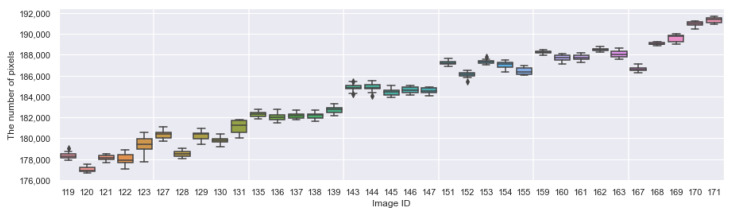
27 August–2 September.

**Figure A4:**
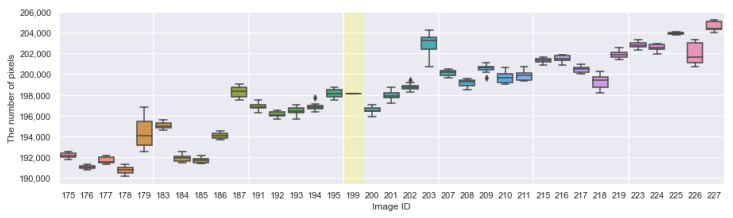
3–9 September.

**Figure A5:**
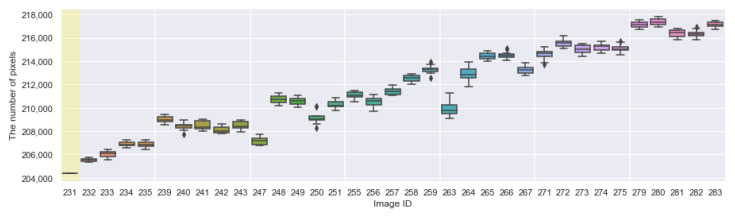
10–16 September.

**Figure A7:**
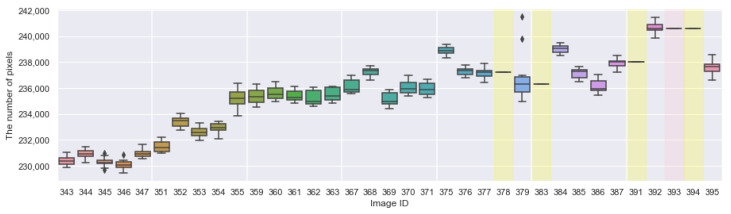
24–30 September.

**Figure A8:**
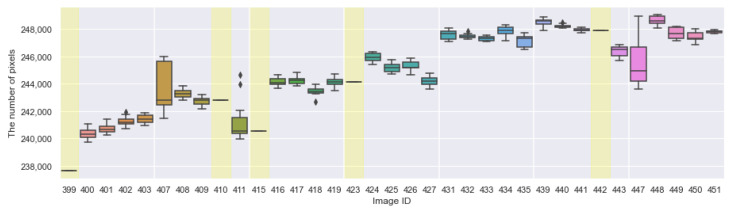
1–7 October.

**Figure A9:**
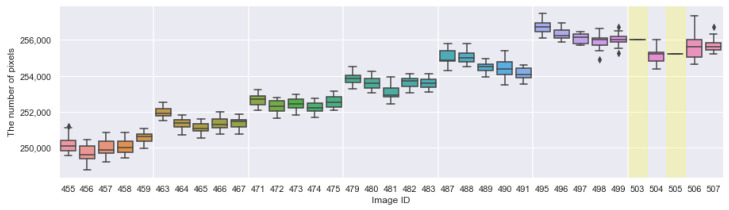
8–14 October.

## Data Availability

Our trained neural network CROP and the related programs are available on GitHub (https://github.com/MotohisaFukuda/CROP, accessed on 20 October 2021). Some of the images used for the qualitative analysis in this paper came from the image gallery organized by United States Department of Agriculture, Agricultural Research Service (https://www.ars.usda.gov/oc/images/image-gallery, accessed on 20 October 2021). Data_Fruit the training dataset in this project consists of images downloaded from Pixabay (https://pixabay.com, accessed on 20 October 2021).

## Appendix A. The Boxplots of the Modified Daytime Data

The boxplots of the modified data are found below. The medians were used for the plot in Figure 19. Yellow indicates removal for high CV, and red the manual removal.
